# Healthy shopper? Blood pressure testing in a shopping centre Pop-Up in England

**DOI:** 10.1186/s12889-018-6370-0

**Published:** 2019-01-23

**Authors:** Laura A. Edwards, Peter Campbell, Deanna J. Taylor, Rakhee Shah, David F. Edgar, David P. Crabb

**Affiliations:** Division of Optometry and Visual Science, School of Health Sciences, City, University of London, Northampton Square, London, EC1V 0HB UK

**Keywords:** Hypertension, Blood pressure, Case finding, Screening, Public health

## Abstract

**Background:**

Improving detection of elevated blood pressure (BP) remains a public health need. We present results from a Pop-Up health check stationed in shopping centres in England. We hypothesise the rate of case detection is related to measurable ‘unhealthiness’ of the shopping centres.

**Methods:**

A Pop-Up health check was sited in four and three shopping centres sampled from the top ten *unhealthiest* and top 15 *healthiest* shopping regions respectively, following a report ranking towns/cities based on their unhealthy and healthy retail outlets. On one day in each shopping centre, people were approached and consented to BP testing. Outcome measure was people flagged with BP ≥ 140/90 mmHg (cases).

**Results:**

We detected 45 (22.6%) and 20 (13.1%) cases from testing 199 and 152 adults in the *unhealthy* and *healthy* locations respectively (relative risk 1.72; 95% confidence interval: 1.06 to 2.78). A measure of *unhealthy* retail outlets (e.g. fast-food outlets) within each shopping centre was associated with detection rate (R^2^ = 0.61; *p* = 0.04).

**Conclusion:**

An association exists between cases of suspect hypertension found in a health check Pop-Up and measured ‘unhealthiness’ of the shopping centre site. Results hint at strategies for public testing of BP, potentially in the context of reducing health inequalities.

**Electronic supplementary material:**

The online version of this article (10.1186/s12889-018-6370-0) contains supplementary material, which is available to authorized users.

## Background

Systemic hypertension is a major cause of mortality and morbidity despite availability of preventive interventions [1, 2]. More than one in four adults in England have hypertension although many are unaware of it. Moreover, half the adult population in England simply do not know their blood pressure (BP) ‘numbers’. Identifying treatable hypertension is cost-effective and Public Health England has called for improvement in detection rates, especially in deprived groups, via outreach testing [3]. For example, it is estimated that people from the most deprived areas in England are 30% more likely than the least deprived to have elevated BP [3].

Retail short-term sales spaces, often referred to as Pop-Ups, are a common sight in shopping centres and other public spaces. Pop-Ups create a temporary retail environment that engages customers and generates a feeling of interactivity [4]. Research evidence suggests retail Pop-Ups increase ‘brand awareness’ and are effective marketing tools [5, 6]. The Pop-Up retail sector is estimated to contribute more than £2 billion per year to the UK economy and large numbers of retail consumers visit Pop-Up shops [7].

In England, BP testing is typically carried out within primary care but other testing opportunities exist, including the National Health Service (NHS) Check Invitation and independent campaigns where testing is initiated directly in communities. To our knowledge, temporary Pop-Up health checks in shopping centres have not been explored and this is the main idea of this study.

Since 2009, adults aged 40–74 years in England have been entitled to an NHS Health Check, a scheme designed to find people with early signs of cardiovascular disease (CVD), kidney disease, type 2 diabetes or dementia [8]. Adults within the age range with no known pre-existing conditions are invited to attend a health check every 5 years. These checks are community based; they are delivered via general medical practices, community pharmacies or another community-based provider [9]. An individual’s CVD risk is predicted by taking into account their sociodemographic characteristics, cholesterol, blood pressure, history of smoking and family history [10]. Those found to be at a higher risk of CVD are placed on a ‘high risk’ register and offered annual reviews. Although a primary aim of the NHS Health Check was to reduce health inequalities, uptake of these checks is relatively low, with those at highest risk of CVD more likely not to attend [11]. A recent systematic review of the delivery and impact of the NHS Health Check concluded that published attendance, uptake, and prescribing rates are all lower than originally anticipated, and data on impact are limited, with very few studies reporting the effect of attendance on health-related behaviours [12]. Moreover, this study also found the uptake of the NHS Health Check to be relatively lower in those living in the most deprived areas. Other studies have also questioned the practicalities [13] and clinical effectiveness of this national prevention programme [14]. Hence, proactively seeking out people at risk of CVD in the community remains an unmet public health need.

By investigating the concentration of businesses and retail outlets that may reflect the state of peoples’ health in cities and towns, the Royal Society for Public Health (RSPH) published a league table of *healthy* and *unhealthy* shopping regions in the UK [15]. Measures were based on, for example, the presence of tanning salons, fast-food restaurants, bookmakers and payday lenders as indictors of ‘unhealthy’ retail outlets. The RSPH published these results as part of their initiative to reduce the negative influences on shopping areas. For example, the report aimed to promote the idea of a public health criterion to be a condition of licensing for all types of business.

We visited shopping centres in different locations in England to test a series of hypotheses on public engagement with our Pop-Up health check using the RSPH report results as a sampling frame. Primarily, our Pop-Up offered a free check for elevated intraocular eye pressure, a risk factor for the eye disease glaucoma [16] and this is the subject of another report. On 50% of the testing days, we offered a free BP check to investigate how this might encourage engagement with the eye health assessment. From this, we took the BP data to develop the hypothesis that the proportion of suspected cases of hypertension detected would vary by shopping centre location. More precisely, for this report, we hypothesised test results might be associated with a measure of the ‘unhealthiness’ of the shopping centre.

## Methods

**Fig.1:**
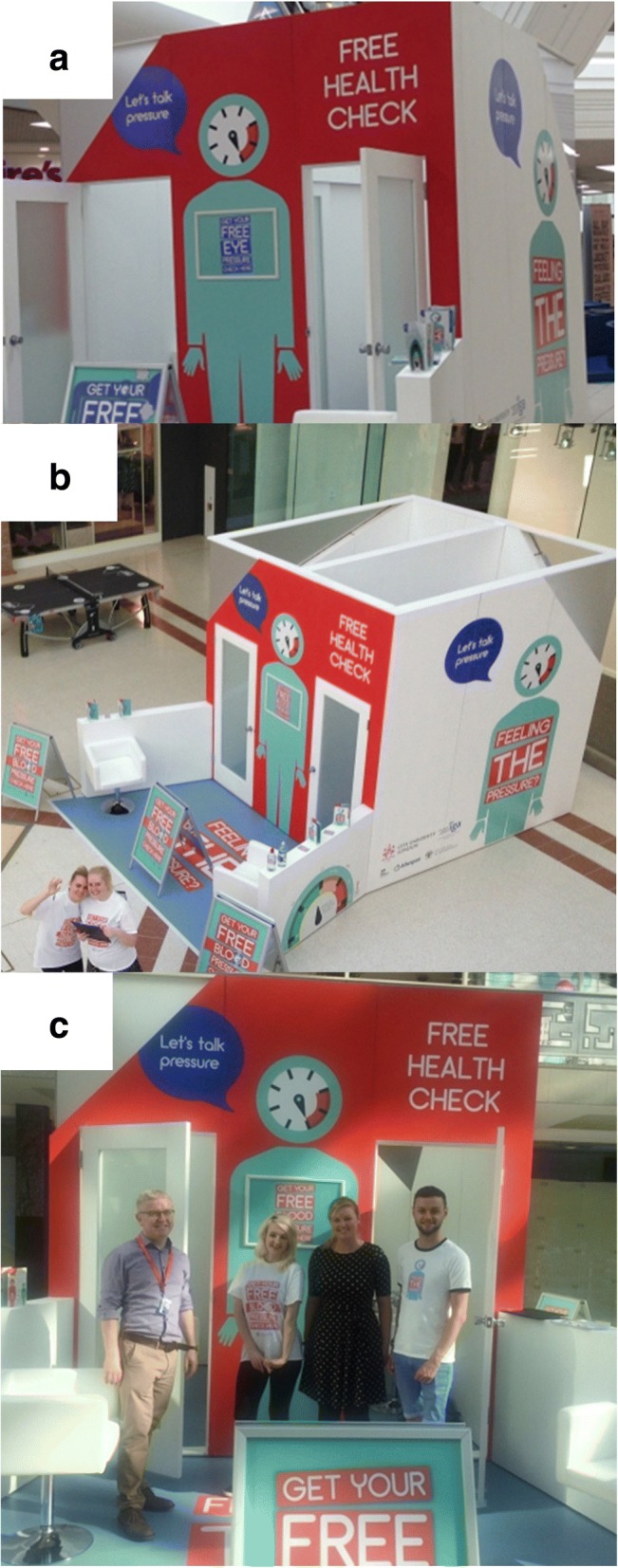
“Feeling the Pressure” Pop-Up pictured in a shopping centre atrium in (**a**) Bristol (The Galleries, BS1 3XD), (**b**) Coventry (Lower Precinct, CV1 1NQ), (**c**) Cambridge (The Grafton Centre, CB1 1PS). The Pop-Up was also located (not pictured) in Preston (St Georges, PR1 2TU), Stoke-on-Trent (Intu Potteries, ST1 1PS), Northampton (Weston Favell, NN3 8JZ) and Nottingham (Intu Broadmarsh, NG1 7LB). (People pictured are assistants and team members.)

This was a prospective, cross-sectional study designed to capture BP measurements in people in the community using a Pop-Up in shopping centres across England. Our “Feeling the Pressure” Pop-Up was designed for use in covered areas (Fig. 1). The Pop-Up comprised two private testing areas and an open reception space designed to engage the public. The Pop-Up was assembled for two consecutive working days in different shopping centres across England during August 2016. All testing was performed by two optometrists assisted, in recruitment and administration, by assistants comprising volunteer undergraduate and postgraduate students. Primarily, the Pop-Up offered a free check on eye health. Additionally, on one of the two testing days in each centre the Pop-Up also offered a free BP check. In this report, we consider the BP data only.

Our sampling of shopping centre locations attempted to follow the schema described in a recently published RSPH (www.rsph.org.uk) report ranking town/city centres based on the number and impact of the most harmful or *unhealthy* and the most beneficial or *healthy* businesses [15]. Four shopping centres were sampled from the reported top ten *unhealthiest* towns/cities. These were (with RSPH report ranking for most unhealthy town/city out of *n* = 70 towns/cities and initials of testing optometrists in parenthesis) in Preston (#1; LAE & DJT); Coventry (#3; LAE & PC); Northampton (#5; LAE & RS); Stoke-on-Trent (#9; LAE & DJT). Three shopping centres were sampled from the bottom of the list and, by implication, were amongst the top 15 *healthiest* towns/cities. These were in Nottingham (#55; LAE & RS); Bristol (#61; LAE & PC); Cambridge (#64; LAE & DJT). The sampling was purposeful but restricted by availability of shopping centres during the study period and logistics.

We determined our own unhealthy retail outlet score for each shopping centre based on a modified version of that used in the RSPH study. On the day of testing, the lead author (LAE) and two Pop-Up assistants surveyed all the retail outlets within the shopping centre by counting the number open to shoppers on the day of testing. They then established the retail outlets within each shopping centre which could be classed as one of four types: either a fast-food takeaway, a bookmaker, a tanning salon or a payday loan business, following the guidelines and descriptions used in the RSPH study. If there was any ambiguity about the nature of the retail outlet, as was the case for some counted as fast-food takeaways, then the notes (including photographs) collected on the day were reviewed by all authors and a consensus decision made. The number of these outlets as a proportion (percentage) of all retail outlets open on the day of testing within the shopping centre was then calculated to be our simple surrogate measure for the *unhealthiness* of the shopping centre. There are, however, some important differences between our own unhealthy retail outlet score and the one used in the RSPH study. In the latter, different scores, or weights, were ascribed to different types of retail outlet based on being negatively health prompting. The RSPH study also ascribed scores to retail outlets it defined as being positively health promoting.

People in each shopping centre were approached and consented to BP testing. Our approach to shoppers was opportunistic and did not allow for estimates of accurate response rates. We aimed to recruit people > 40 years but did not reject approaches from younger people. The examination was free. For this study, contact details, age and details of the participant’s General Practitioner (GP) were recorded. A short medical history was taken. BP was measured using a Panasonic EW3106 monitor (Panasonic; Osaka, Japan); a device approved by the British Hypertension Society (BHS; www.bhsoc.org) which meets the European Society of Hypertension accuracy levels [17]. All four optometrists (LAE; DJT; PC; RS) had training in good practice and competency for measuring BP under guidance of a lecturer in nursing at City, University of London. BP was measured after five minutes resting, with the participant sitting with their left arm supported at heart level. Care was taken to use an appropriate size cuff bladder. Everyone tested was given his or her BP measurement recorded on an information leaflet about BP specifically designed for this study. The outcome measure for this study was people with a BP of ≥140/90 mmHg on repeat testing. (For the repeat BP testing, we followed the NICE Clinical Guideline CG127 [18]. So, for example, if the second BP measurement was substantially different from the first, then a third measurement was taken with the lower of the last two measurements recorded.) For this report, we define these people as cases. For these cases, we also sent a referral note to their GP. Some participants were already aware of their elevated BP or were on BP-lowering treatment; these participants were still measured and classified as cases if their BP was ≥140/90 mmHg. Following relevant national guidelines, people with BP of > 180/110 mmHg were counselled on the urgency of their referral, reflected in a different GP letter in addition to a follow-up phone call to the individual. Anyone with BP > 210/120 mmHg was to be immediately directed to an Accident and Emergency department, facilitated by the Pop-Up assistants.

The research was approved by a university ethics committee and monitored by an advisory group comprising different stakeholders including members of the public. Written informed consent, according to the tenets of the Declaration of Helsinki, was obtained from each participant prior to examination. All participants were told the examination might give them useful information about their general health, but they were also advised that the Pop-Up examination was no substitute for an assessment by their GP; this information was conveyed verbally and stated clearly in the participant information sheet. Moreover, all participants were given an easy-to-read purpose written information leaflet (see Additional file 1) which was subject to scrutiny and approval by the advisory group and ethics committee. Data were recorded both manually and using a tablet computer on the day of testing. All information was anonymised, then subsequently transferred and stored onto a secure database held at the university.

Analysis of data centred on the proportion of cases as compared to all those who consented to their BP being measured in shopping centres pooled across the four *unhealthy* and three *healthy* locations. We also tested for univariate association between our shopping centre *unhealthiness* measure and the proportion of cases across the seven shopping centres. All data analysis was carried out in Microsoft Excel and R (www.R-project.org).

## Results

In total, 199 people (48% male) were examined in the four *unhealthy* locations over four days and 152 people (52% male) were examined in the three *healthy* locations over three days. The two samples examined had almost identical age distributions: median (10th, 25th, 75th, 90th percentile) age was 56 (28, 41, 70, 76) and 56 (27, 40, 68, 75) years for the *unhealthy* and *healthy* locations respectively.

Number of people tested and cases (people flagged with a BP of ≥140/90 mmHg on repeat testing) identified at each shopping centre is given in Table 1. For our main outcome we detected 45 (22.6%) and 20 (13.1%) cases in the *unhealthy* and *healthy* locations respectively. The difference in the proportion of cases indicates a statistically significant relative risk (1.72; *p* = 0.03). These results suggest that a person tested in an *unhealthy* shopping region is 72% (95% confidence interval: 6 to 278%) more likely to be tested positive as a case than a person tested in a *healthy* shopping region.

**Table 1:** Number of people tested and cases identified in each shopping centre with RSPH report ranking for most unhealthy town/city out of *n* = 70 towns. In the column of cases, the figure in parenthesis is the number of people who were already aware of having elevated BP, or self-reported some history of issues with elevated BP

Shopping centre region	Number of people tested	Cases: BP ≥140/90 mmHg	People: BP > 180/110 mmHg
Preston (#1)	53	12 (8)	2 (1)
Coventry (#3)	43	4 (1)	0 (0)
Northampton (#5)	58	19 (10)	2 (2)
Stoke-on-Trent (#9)	45	10 (2)	0 (0)
Totals	*199*	*45 (21)* *22.6%*	*4 (3)*
Nottingham (#55)	44	7 (4)	0 (0)
Bristol (#61)	55	6 (3)	0 (0)
Cambridge (#64)	53	7 (4)	0 (0)
Totals	*152*	*20 (11)* *13.1%*	*0 (0)*

The difference in the proportion of cases indicates a statistically significant relative risk (1.72; *p* = 0.03)

Around one half of all cases were already aware of having elevated BP, or self-reported some history of issues with elevated BP (Table 1). Four people recorded BP > 180/110 mmHg and these were all recorded in the shopping centres in the unhealthy locations. There were no instances of a person having a BP > 210/120 mmHg.

**Fig. 2:**
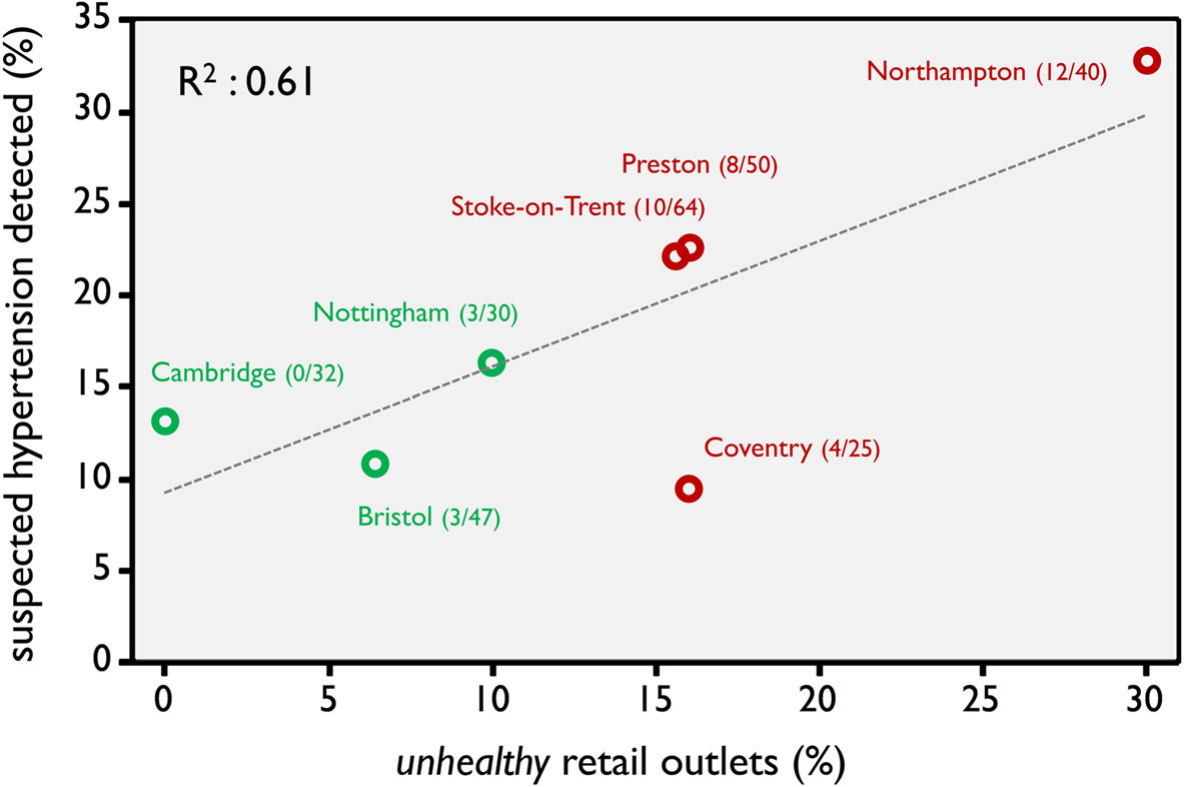
Relationship between percentage of cases detected and percentage of *unhealthy* retail outlets (fast-food takeaways; bookmakers; tanning salons; payday loan businesses) within shopping centres. Fractions in parenthesis are (number of unhealthy retail outlets/total number of outlets) for each shopping centre

As would be expected, our local measure of percentage of *unhealthy* retail outlets within each shopping centre aligned closely with the observations in the RSPH survey. For example, 34 retail outlets out of a total of 179 (19.0%) were identified to be either a fast-food takeaway, a bookmaker, a tanning salon or a pay-day loan business in our four shopping centres sampled from unhealthy locations. (See Fig. 2 for numbers by individual shopping centre.) This estimate was significantly lower (6/109; 5.5%) in our three shopping centres in healthy regions. This equates to more than a threefold difference (95% confidence interval: 1.5 to 7.9). One shopping centre in Cambridge had none of these ‘unhealthy’ retail outlets. Conversely more than one quarter of the retail outlets in the shopping centre in Northampton were, remarkably, either a fast-food takeaway, a bookmaker, a tanning salon or a payday loan business.

Our local measure of percentage of *unhealthy* retail outlets within the shopping centre was associated with the detection rate of cases and this was statistically significant (R^2^ = 0.61; *p* = 0.04; Fig. 2).

## Discussion

By surveying the retail outlets that may reflect the state of peoples’ health in cities and towns, the RSPH published a league table of *healthy* and *unhealthy* shopping regions [15]. In our study, we found a person volunteering to be tested in a heath check Pop-Up in one of the *unhealthy* regions defined by the RSPH report is more likely to have suspected or diagnosed elevated BP compared to an age-similar person tested in a *healthy* town/city and this is our principal finding. Moreover, a measure of *unhealthy* retail outlets within a shopping centre was significantly associated with detection rate of suspected or diagnosed elevated BP in our sample. In other words, the proportion of fast-food takeaways, bookmakers, tanning salons and payday loan outlets within a shopping centre is related to the number of cases of elevated BP in people volunteering to be examined as part of a Pop-Up health check conducted in the shopping centre. Our findings add to the knowledge of potential methods of assessing people at risk of hypertension and reveal an interesting link to location of testing.

Relationships between shopping environment and health have been documented in the research literature. For example, data from a population health survey of 2900 adults was linked with geographic measures of access to food retailers in Edmonton in Canada. Results indicated the ratio of fast-food restaurants and convenience stores to grocery stores and produce vendors near people’s homes was related to likelihood of being obese [19]. Other research from the United States supports the claim that access to fast-food restaurants is associated with obesity among adults [20], excess weight gains over time [21] as well as insulin resistance [22]. There is also good evidence to show that excessive use of fast-food restaurants is associated with higher BMI in children in the UK [23]. The RSPH report highlighted the importance of ensuring retail areas encourage healthy lifestyles and suggested businesses such as fast-food outlets, betting shops, and payday lenders should be unable to cluster in areas of high deprivation [15]. Therefore, we believe our measure of an unhealthy shopping environment to be a reasonable one. For example, payday lending is a contemporary public health concern because of the vulnerability of the populations using these lenders and the documented detrimental effect that financial difficulties can have on mental and physical health [24]. Moreover, there is a strong association between a profusion of betting shops and problems with gambling and poor health indices [15]. Sunbed use is associated with a significant increase in risk of melanoma [25] and at least one systematic review has shown that the typical sunbed user is more likely to have an unhealthy diet, smoke and drink alcohol more frequently than a non-user [26].

To our knowledge the idea of a Pop-Up health check for BP, set in a shopping centre, has not been previously explored. Pop-Up clinics have been proposed and examined for HIV testing [27] and there are several reports on the effectiveness of mobile health clinics designed to raise awareness and screen for a wide variety of conditions, such as, colorectal cancer [28], paediatric eye disorders [29] and general health in vulnerable populations [30]. Moreover, mobile health clinics are widely used in the US and, for example, one extensive study has found them to be effective for screening for suspect hypertension [31]. Interestingly, using the Pop-Up concept to improve public health has recently been extended to the idea of temporary urban Pop-Up parks designed to solve the limited access to public physical activity recreation spaces many urban residents face [32]. Our novel idea of a Pop-Up health check in a shopping centre certainly fits with a recent call by NHS England and Public Health England, urging council public health teams to organise BP check opportunities in public places [33].

Around one half of all cases reported in this study were already aware of having elevated BP, or self-reported some history of issues with elevated BP. The effect we detected between the *unhealthy* and *healthy* shopping regions could therefore be explained by differences in undiagnosed hypertension/suspected hypertension or be explained by differences in possible poor control of known hypertension/suspected hypertension; as likely, the effect could be explained by a combination of the two factors. Overall numbers were not substantial enough for us to make a distinction between these two factors. Yet this distinction is important. One centres on medication adherence and long-term management issues, whilst the other centres on detection. Both have substantial public health importance and their relationship with socioeconomic status should be studied further, as suggested by other reports [34].

There are some critical limitations to our observations. For example, there are ethnic differences in the prevalence of hypertension but we did not record or report our participants’ ethnicity. Prevalence of hypertension is raised in South Asian, Afro-Caribbean, and West African people in England and ethnicity is an important consideration in assessing BP measures in community-based studies [35]. We therefore cannot comment on a bias that might be introduced by some areas having higher prevalence of different ethnic groups compared to others. Similarly, a bias in our results may have been introduced by differences in levels of obesity between ‘healthy’ and ‘unhealthy’ locations or differences in ‘white coat’ hypertension whereby the clinical setting precipitates artificially elevated BP due to increased patient anxiety [36].

Furthermore, a diagnosis of elevated BP cannot be made from measurements at a single point in time. One author (LAE) performed most, but not all of the testing so our results might be limited by the use of different assessors. Nevertheless, other discrepancies in measurement from, for example, failure to position participants and their arms consistently would have been minimised by the identical testing environment afforded by the purpose-built Pop-Up. An unavoidable limitation of the results from our case finding exercise arises from only being able to assess individuals interested in having their BP measured. Moreover, we originally aimed to test only people who were > 40 years but the Pop-Up generated a lot of interest and we examined younger people too; consequently, around one quarter of our participants were younger than 40 years. Our study design meant our results are limited to observational associations. Moreover, our study did not have a longitudinal element where we could, for example, follow-up the suspected cases. In addition, whilst our unhealthy retail outlet score is based on a previous report [15] it is very much a surrogate measure and has not been validated in other studies.

There is more to understand about different ways to case-find suspect hypertension. A proven effective route is to create easily accessible testing opportunities such as in community settings or the workplace [37] [38]. There are other advantages to screening for hypertension away from a ‘white coat’ medical environment [36]. Of course, these forms of testing are still restricted to individuals who volunteer to have their BP measured and current evidence is insufficient to recommend specific approaches for community-based case finding for elevated BP [39]. Nevertheless, our study contributes a modest example of a new approach to assessing BP outside primary care. The health check Pop-Up also offers a way of educating the public about hypertension and BP. Our results also imply that an in situ public health check might benefit from a targeted strategy, not seen in current approaches [40]. We assessed 351 people in only 7 days of testing, a remarkable number given the tests of eye health carried out on the same day.

## Conclusions

In conclusion, we demonstrate an intriguing relationship between detecting people with suspected elevated BP and the type and location of the shopping centre they were visiting. We speculate our results hint at strategies for targeted outreach testing and screening of BP that should be the subject of further investigation, potentially in the context of reducing health inequalities.

## Additional file

Additional file 1: Blood pressure leaflet. (PDF 1111 kb)

## Abbreviations

BP: Blood pressure
GP: General practitioner
NHS: National Health Service
RSPH: Royal Society of Public Health
UK: United Kingdom
