# Supplementary material for: Healthy shopper? Blood pressure testing in a shopping centre Pop-Up in England
Source: BMC Public Health. 2019 Jan 23;19:42. doi: 10.1186/s12889-018-6370-0 (PMC6343246; doi:10.1186/s12889-018-6370-0)
Supplement: Supplementary file 1 — Blood pressure leaflet. (PDF 1111 kb) [file 12889_2018_6370_MOESM1_ESM.pdf]

And today's magic number is...

On .... your blood pressure

was .... mm/Hg

The top number
Systolic blood pressure. The highest pressure – when your heart pumps.

The bottom number
Diastolic blood pressure. The lowest pressure – when your heart relaxes.

So, what does that mean?

Your ideal blood pressure depends on lots of things, including your age.

For more info visit
www.bloodpressureuk.org

Give those blood vessels some love

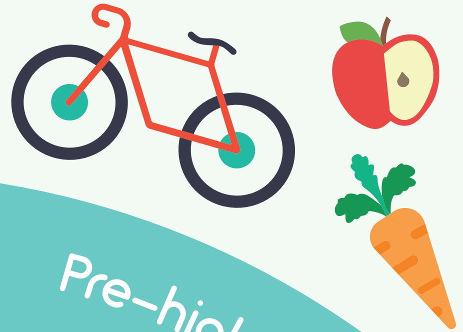

1. Eat less salt
2. Eat more fruit and veg
3. Keep a healthy weight
4. Drink less alcohol
5. Get more active

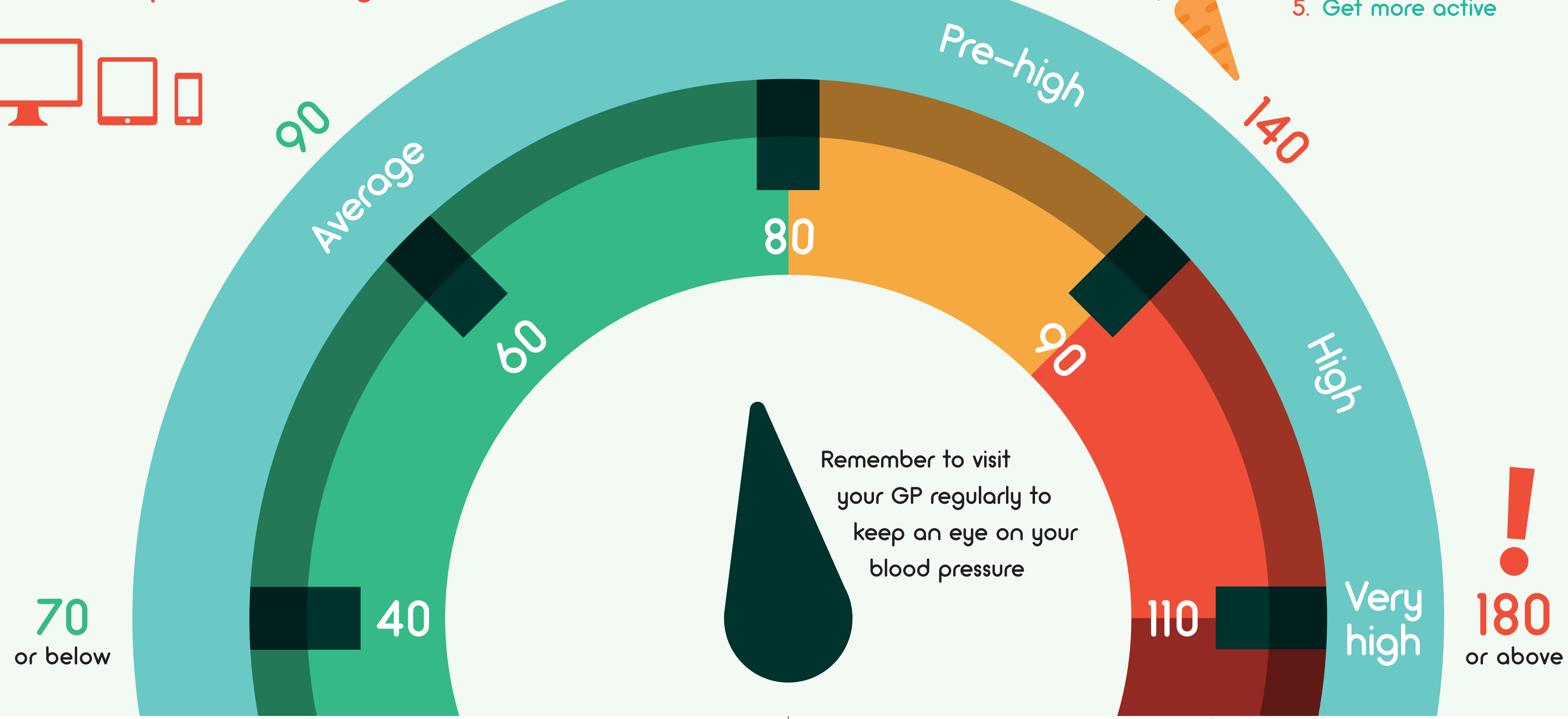

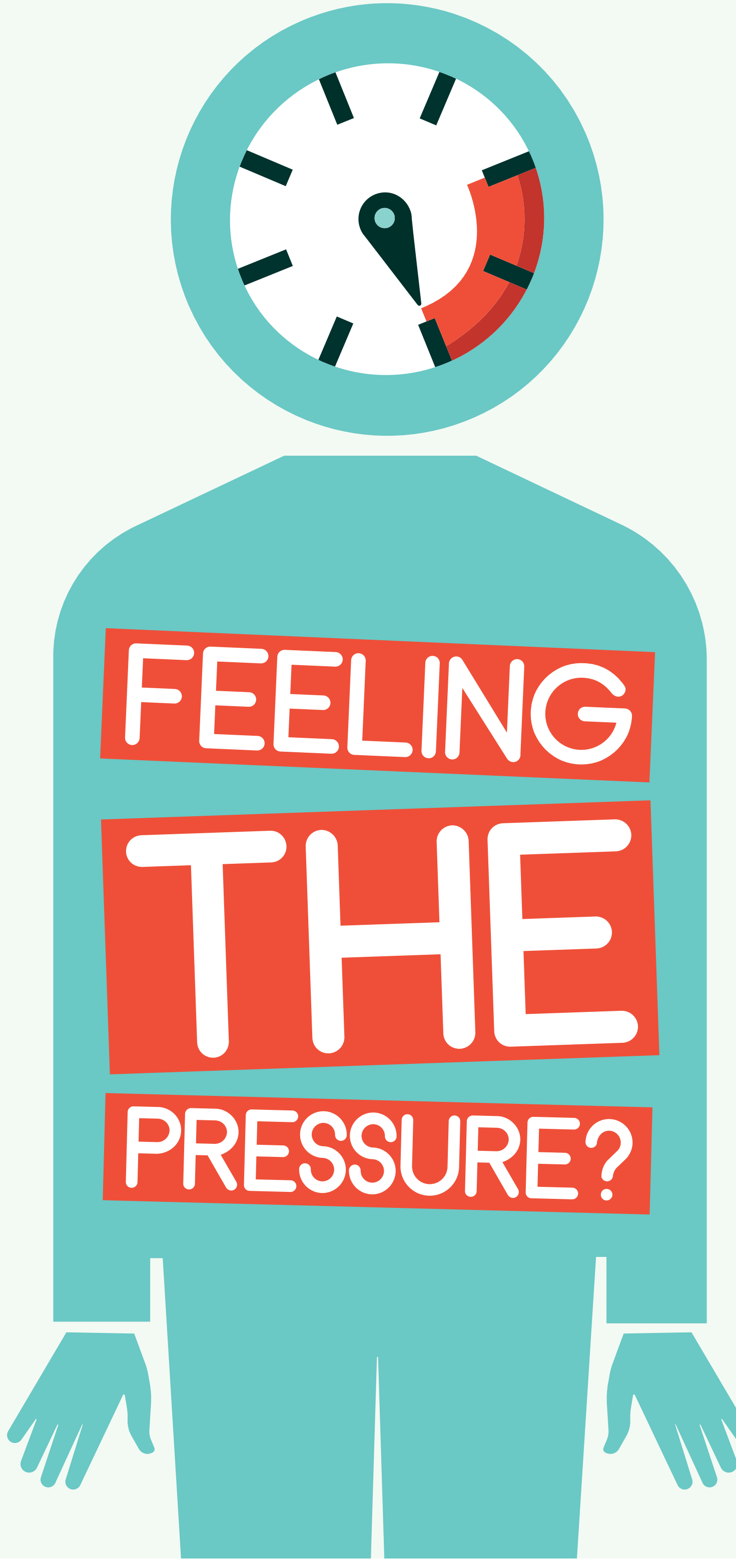

Information on blood pressure

Developed by City University London to support a pop-up pilot study investigating health in the high street

City University London
The College of Optometrists
Fight for Sight – The Eye Research Charity
International Glaucoma Association iga – The Charity for People with Glaucoma, Established 1974

City Uni Leaflet A5_6pp_BP.indd 1-3
01/06/2016 18:26

That funny old thing called life

Sometimes we all feel life's pressures. But there are some really important pressures in your body that you don't feel. This leaflet is all about one of those pressures.

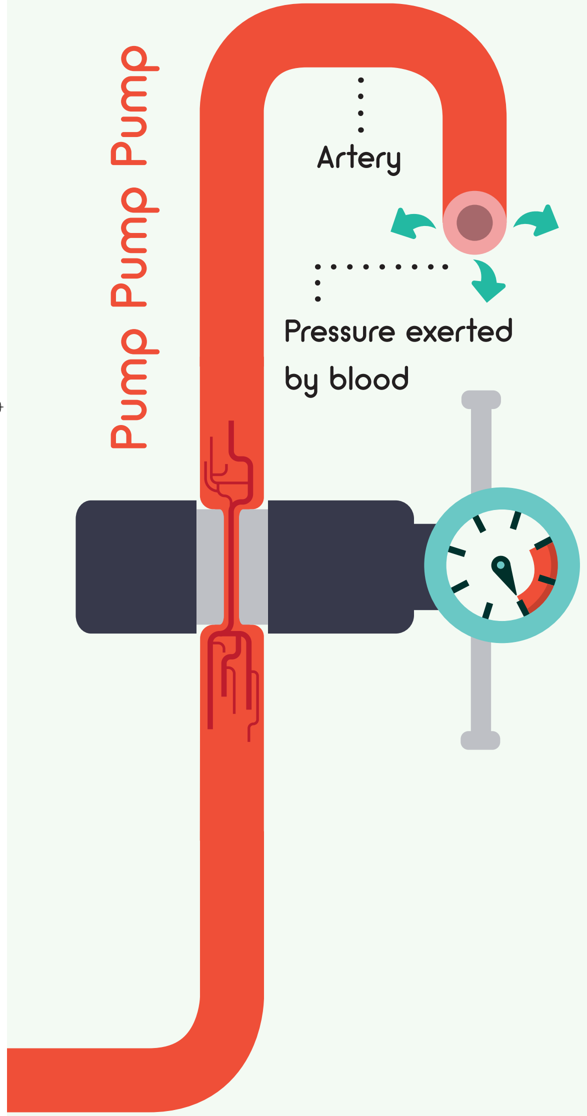

When your heart beats, it pumps blood through your blood vessels. The force of the blood pushing inside your vessels is your blood pressure.

Crankin' up the pressure

Blood pressure changes over time. It's affected by things like what you eat and drink, and how much exercise you do.

If blood pressure gets higher than it should be, it puts strain on your blood vessels and heart.

Is it serious, doc?

High blood pressure can mean...

High blood pressure can damage your blood supply, blood vessels and organs. It can seriously affect your general health, and could mean heart attack or stroke.

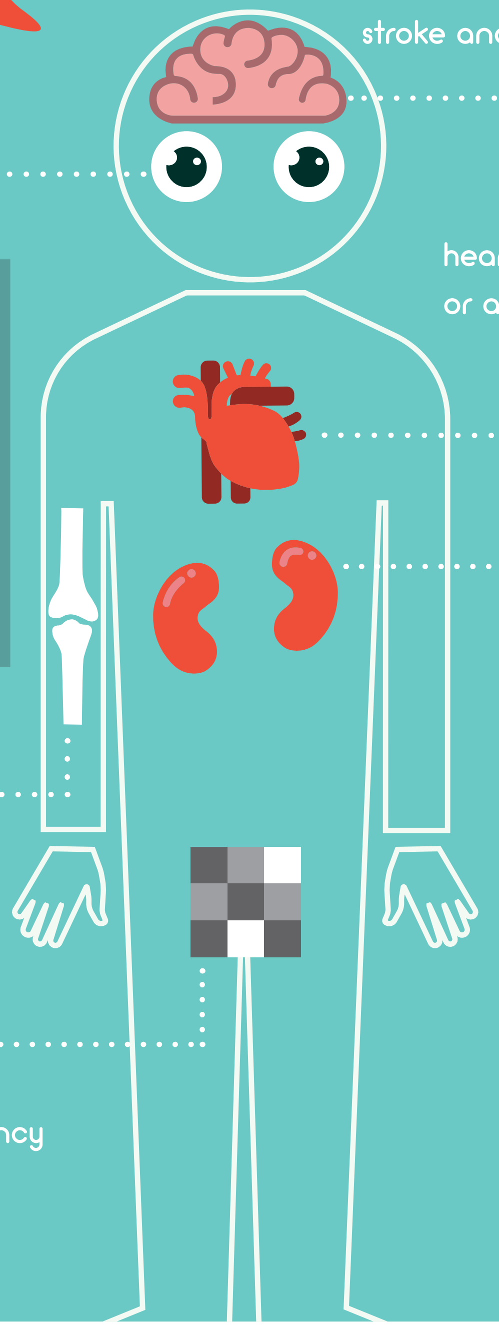

Check it out

Even if blood pressure gets a little bit higher, your risks can get a lot bigger. The only way to know your blood pressure is to have it checked regularly.

A blood pressure reading has two numbers – one on top of the other like this:

$$\frac{120}{80}\ \text{mmHg}$$

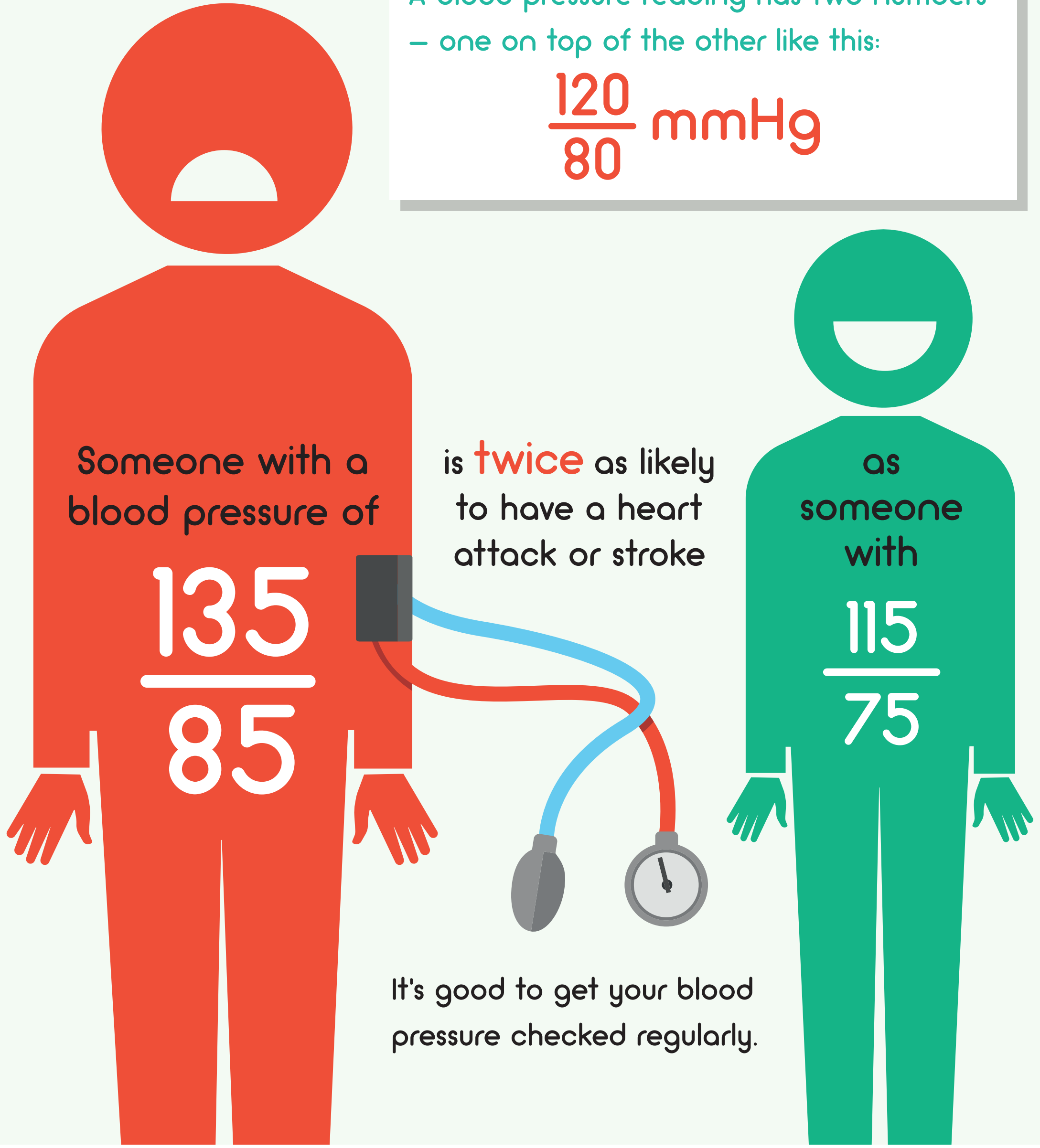

It's good to get your blood pressure checked regularly.

City Uni Leaflet A5_6pp_BP.indd 4-6 01/06/2016 18:26
